# The Impact of Thyme and Rosemary on Prevention of Osteoporosis in Rats

**DOI:** 10.1155/2019/1431384

**Published:** 2019-03-31

**Authors:** Amr S. Elbahnasawy, E. R. Valeeva, Eman M. El-Sayed, I. I. Rakhimov

**Affiliations:** ^1^Department of Bioecology, Hygiene and Public Health, Institute of Fundamental Medicine and Biology, Kazan Federal University, Kazan, Russia; ^2^Department of Nutrition and Food Sciences, National Research Centre, Dokki, Giza, Egypt

## Abstract

Osteoporosis poses an important public health problem which affects millions of people worldwide. There is a direct link between calcium deficiency in diet and induction of osteoporosis and bone loss. The current study was conducted to evaluate the protective effect of thyme (*Thymus vulgaris* L.) and rosemary (*Rosmarinus officinalis* L.) against osteoporosis in rats with low calcium intake. Essential oils of rosemary and thyme were analyzed. The experiment was carried out on growing male Sprague–Dawley rats; the experimental animals were divided into 5 groups: 1, control negative was fed standard balanced diet; 2, control positive was fed balanced diet with low calcium level (L Ca) (Ca 0.1% w/w); 3, (L Ca) + thyme powder (5% w/w); 4, (L Ca) + rosemary powder (5% w/w); 5, (L Ca) + orally administration with CaCO_3_ (27 mg/kg body weight). Blood samples were collected for different biochemical analyses in plasma (calcium (Ca), phosphorus (P), magnesium (Mg), tumor necrosis factor-alpha (TNF-*α*), C-reactive protein (CRP), malondialdehyde (MDA), parathyroid hormone (PTH), C-terminal telopeptide (CTX), and 1,25-(OH)2-vitamin D3). Femur mass, length, and bone mineral density (BMD) were recorded, and histopathological studies for femurs were examined. Low-calcium diet induced osteoporotic changes in positive control rats (decrease in Ca, vitamin D3, and BMD and increase in CTX, PTH, TNF-*α*, CRP, and MDA). Supplementation with thyme and rosemary inhibited significantly the development of bone loss, increased Ca and vitamin D3 in plasma, improved BMD, and also prevented the inflammation and oxidative stress (improved TNF-*α*, CRP and MDA) compared to the positive control. The histopathological examination of treated groups showed an improvement in bone histology and protection against bone loss. However, thyme powder showed more effective impact than rosemary. Our study demonstrates that thyme and rosemary effectively mitigated calcium deficiency-induced bone loss and maybe considered as promising candidates for preventing bone resorption and osteoporosis.

## 1. Introduction

Osteoporosis is a skeletal disease described by a decrease in bone mass and bone mineral density in addition to bone degradation, which may increase bone fracture risk and skeleton frailty and induce dangerous difficulties [1]. In 2010, more than 99 million persons in USA were estimated to have osteoporosis or low bone mass, and this number is expected to increase by 19% by 2020 and by 32% by 2030 [2]. Calcium is an essential component in bone formation and is considered as the key element of hydroxyapatite, and its use as a therapy for bone resorption is evaluated [3]. Improving the dietary intake of calcium is a nutritional target to prevent osteoporosis [4].

Bone resorption sorely affects public health, and there is no effective and secure way to restore lost bone; many pharmacological drugs, such as denosumab, risedronate, alendronate, and bisphosphonates, are used for treatment of osteoporosis. However, the medications most commonly used are not suitable to take and action is often weak [5]. In addition, many patients avoid taking the medicines because of worry about side effects such as nausea, dizziness, sickness, and sore of the gastrointestinal tract [6].

The role of nutrition in bone formation is quite significant. Choosing a balanced diet with high nutritional value can significantly contribute to enhance skeletal health. Appropriate food is an essential element for bone health, resulting in the treatment and prevention of bone diseases [7]. However, many medicinal herbs prevent osteoporosis and treat bone resorption [8, 9]. Polyphenols extracted from plants attract big attention due to their antimicrobial and antioxidant activities [10]. Phenolic compounds have a valuable free-radical scavenging property, which is determined by their reactivity with other antioxidants compounds and their metal chelating activities [11].

Thyme (*Thymus vulgaris* L.) is a medicinal herb containing many volatile compounds and essential oils and is widely used worldwide and considered to have potential protective impact against bone loss [12]. The essential oils are nontoxic and biodegradable compounds with antimicrobial activity and without side effects or intestinal troubles after eating [13]. Thyme essential oil consists mainly of thymol, carvacrol, *γ*-terpinene, and *β*-caryophyllene [14]. It has been confirmed that the biochemical impacts of thymus vulgaris are primarily due to the existence of phenolic compounds, especially thymol and carvacrol [15].

Rosemary (*Rosmarinus officinalis* L.) is supposed to be one of the most important herbs that has many medical benefits, the main volatile compounds are 1,8-cineole, camphor, *α*-pinene, and *α*-terpineol [16]. Rosemary is widely used as a flavoring agent in food industries [17]. However, rosemary has potential pharmaceutical benefits in prevention and treatment of many public health issues [18–20]. The aim of the present work is to investigate the inhibitory impact of thyme and rosemary against osteoporosis in rats with calcium deficiency.

## 2. Materials and Methods

### 2.1. Plant Materials

Fresh dry herbs of rosemary (*Rosmarinus officinalis* L.) and thyme (*Thymus vulgaris* L.) were obtained from the Medicinal and Aromatic Plant Unit, National Research Centre, Cairo, Egypt. The dried leaves were ground to obtain a fine powder.

### 2.2. Isolation and Analysis of Volatile Compounds of Rosemary and Thyme

Rosemary and thyme were subjected to hydrodistillation for 3 hours using Clevenger-type apparatus for isolation the essential oil in fresh herbs. The obtained oil of each sample was dried over anhydrous sodium sulfate [21]. The collected essential oils were immediately analyzed using gas chromatography (GC) analysis which was performed by using a PerkinElmer autosystem XL equipped with a flame ionization detector (FID). A fused silica capillary column DB5 (60 m × 0.32 mm id) was used. The oven temperature was maintained initially at 50°C for 5 min and then programmed from 50 to 250°C at a rate of 4°C/min. Helium was used as the carrier gas at flow rate 1.1 ml/min. The injector and detector temperatures were 220 and 250°C, respectively. The retention indices (Kovats index) of the separated volatile components were calculated using hydrocarbons (C8–C22, Aldrich Co.) as references.

### 2.3. Experimental Animals and Diets

Thirty-five growing male Sprague–Dawley rats weighing 70 ± 10 g, aged 28 days were obtained from the laboratory animal house, National Research Centre, Egypt. The animals were housed individually in stainless steel cages in a controlled environment (25 ± 2°C, 50–60% relative humidity, and 12-hour light-dark cycle), and the experiment lasted for 8 weeks. The animals had ad libitum access to food and deionized water and were subdivided into five groups (7 rats each) as follows: group 1: control negative (C−) was fed standard balanced diet AIN-93G with normal calcium level (0.5% w/w) according to formula described by Reeves et al. [22]; group 2: control positive (C+) was fed balanced diet with low calcium level (L Ca) (Ca 0.1% w/w) according to Shinoki and Hara [23]; group 3: thyme (T) was given L Ca diet and treated with thyme power (5% w/w) [24]; group 4: rosemary (R) was given L Ca diet and treated with rosemary power (5% w/w) [25]; group 5: oral calcium (O) was orally administered with CaCO3 (27 mg/kg b.w.) and fed L Ca diet [26].

### 2.4. Biochemical Analyses

At the end of experiment, the rats were fasted overnight, blood samples were collected in heparinized tubes, and centrifuged at 2500 rpm under cooling for 15 min to separate the plasma which was subjected to different assays. Plasma calcium (Ca), phosphorus (P), and magnesium (Mg) levels were determined using colorimetric assay kits (BioSystems S.A., Costa Brava, Barcelona, Spain). Plasma tumor necrosis factor-alpha (TNF-*α*) measurements were assayed using an enzyme-linked immunosorbent assay (ELISA) kit by the RayBio® Rat TNF-*α* (RayBiotech, Inc., Norcross, Georgia, USA); the assay of C-reactive protein (CRP) was quantitatively determined by the reagent CRP Turbilatex agglutination assay kit (Spinreact, Girona, Spain); Malondialdehyde (MDA) was determined according to the method of Draper and Hadley [27], based on the reaction of MDA with thiobarbituric acid (TBA). Parathyroid hormone (PTH) was measured in plasma using the ELISA kit (MyBiosource, Inc., San Diego, USA). The C-terminal telopeptide (CTX) was assessed using Plasma Cross Laps One-Step ELISA (Osteometer BioTech, Herlev, Denmark). 1,25-(OH)2-Vitamin D3 was quantitatively determined in plasma using ELISA kit (IBL International GmbH, Hamburg, Germany).

### 2.5. Femur Mass, Length, and Bone Mineral Density

The right femur of each sacrificed rat was dissected out, stripped of all soft tissues, and washed in saline. The femur mass was recorded using electronic balance, and the length was determined with electronic caliper. The bone mineral density of right femur in each group was measured by dual-energy X-ray absorptiometry (DEXA) (Norland XR-600).

### 2.6. Histopathological Examination

Femurs were fixed in 10% formalin in saline solution for 48 h and then decalcified in daily exchanges of EDTA. Each sample was then processed to obtain 6-*μ*m-thick paraffin sections to be stained with heamtoxylin and eosin stains (H&E) for light microscopic histopathological examination according to the method described by Bancroft and Gamble [28].

### 2.7. Statistical Analysis

All values are presented as mean ± standard error (SE); one-way completely randomized analysis of variance (ANOVA) used to compare the difference in values in all experimental groups by Tukey's honestly significant difference (HSD) post hoc test to clarify the significance with significance level (*p* < 0.05) using the CoStat Version 6.451 statistical program (CoHort Software, USA).

## 3. Results

The volatile compounds in the hydrodistilled oil of rosemary were identified and cited with their relative area percentage (Table 1). Among the identified compounds 1,8-cineole was the major component (46.69%) followed by camphor (26.30%), *α*-pinene (5.63%), and *α*-terpineol (5.23%). The separated volatile compounds of thyme were identified and cited with their relative area percentage (Table 2). Thymol was the predominant compound (73.61%) followed by carvacrol (4.97%) and *p*-cymene (4.06%).

As presented in Table 3, supplementation with thyme and rosemary significantly increased plasma calcium levels in both T and R groups (8.92 ± 0.13 and 8.54 ± 0.11) compared to the positive control group (7.57 ± 0.12); on the other hand, magnesium and phosphorus levels had no significant changes among all groups. Concomitantly, the inflammatory biomarkers TNF-*α* and CRP were also estimated as shown in Table 3. Attenuated significant levels of TNF-*α* compared to positive groups (75.24 ± 0.93) were observed in both T and R groups (47.57 ± 0.96 and 44.53 ± 0.98), respectively. However, these levels were significantly higher than normal control (31.60 ± 0.94). The inflammatory level of CRP in the positive control group was significantly higher than that of the normal control (0.85 ± 0.09) and O group (0.82 ± 0.08). Treatment with thyme maintained the level of CRP in group T (0.97 ± 0.08) with no significant difference compared to normal group. As a marker of lipid peroxidation and oxidative stress, the MDA level was assayed (Table 3); malondialdehyde levels were significantly increased in the control positive group (2.42 ± 0.11) compared to both the normal control and O groups (1.33 ± 0.10 and 1.44 ± 0.10); otherwise, treatment with herbs significantly improved these levels in T and R groups (1.92 ± 0.12 and 1.85 ± 0.11).

The bone resorption marker CTX levels also were significantly improved by supplementation in both T and R groups (379.71 ± 5.58 and 392.12 ± 7.24) compared to the positive control (507.92 ± 5.76). On the other hand, the vitamin D3 levels in treated groups T and R was significantly increased compared to positive control (46.84 ± 1.07), but these levels were lower than observed in the control normal group (64.12 ± 1.06) and O group (59.05 ± 1.03) (*p* < 0.05) (Table 3). Furthermore, the PTH level was significantly higher in the control positive group (69.11 ± 1.11) than in both of those intervention groups T and R (50.13 ± 0.96 and 58.87 ± 1.01b), which showed difference from each normal control (45.29 ± 0.92) and oral group (41.21 ± 0.80) (*p* < 0.05).

Femur mass, length, and bone mineral density are shown in Table 4; groups R and O showed no significant changes in femur mass (3.01 ± 0.17 and 3.16 ± 0.07), respectively, compared to the normal control group (3.02 ± 0.07); otherwise, group T showed insignificant value (2.93 ± 0.12) compared to the positive control (2.46 ± 0.11), which recorded the lowest value among all groups group (*p* < 0.05). On the other hand, femur length had no significant differences among all groups. The intervention with herbs caused a significant improvement in BMD in groups T (0.1357 ± 0.0051) and R (0.1305 ± 0.0064) compared to the positive control (0.0981 ± 0.0037); furthermore, both groups T and R had no significant difference compared to the normal control (0.1493 ± 0.0041), but highest BMD level was seen in group O (0.1525 ± 0.0051).

**Figure 1:**
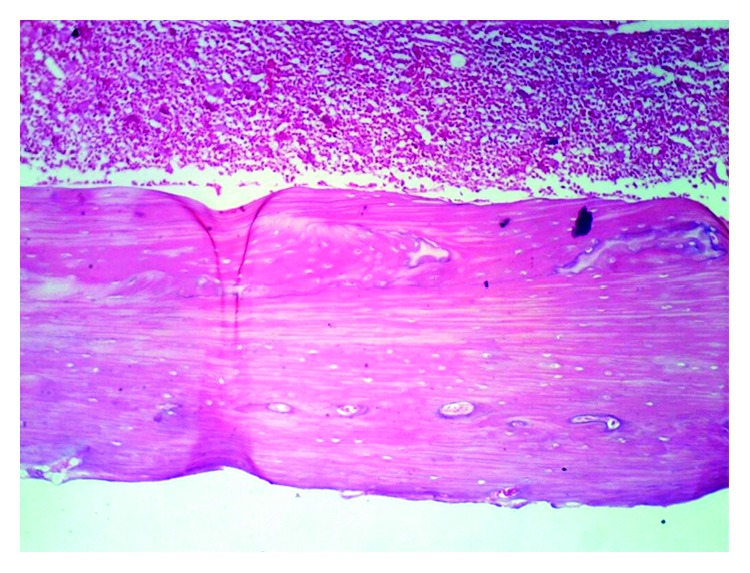
Photomicrograph of bone of rat from the thyme group (T) showing no histopathological changes. *Note*. Normal bone cortex (H&E X 100).

**Figure 2:**
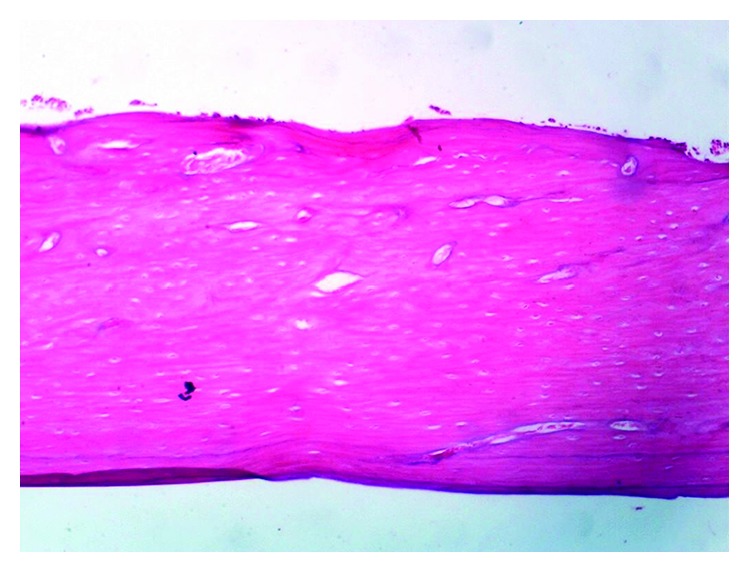
Photomicrograph of bone of rat from the normal control group (C−) showing no histopathological changes. *Note*. Normal bone cortex (H&E X 100).

**Figure 3:**
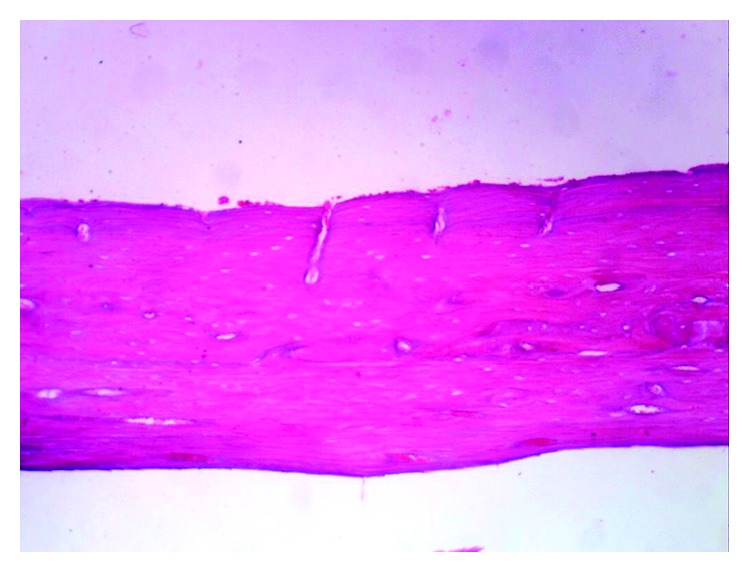
Photomicrograph of bone of rat from oral calcium group (O) showing no histopathological changes. *Note*. Normal bone cortex (H&E X 100).

**Figure 4:**
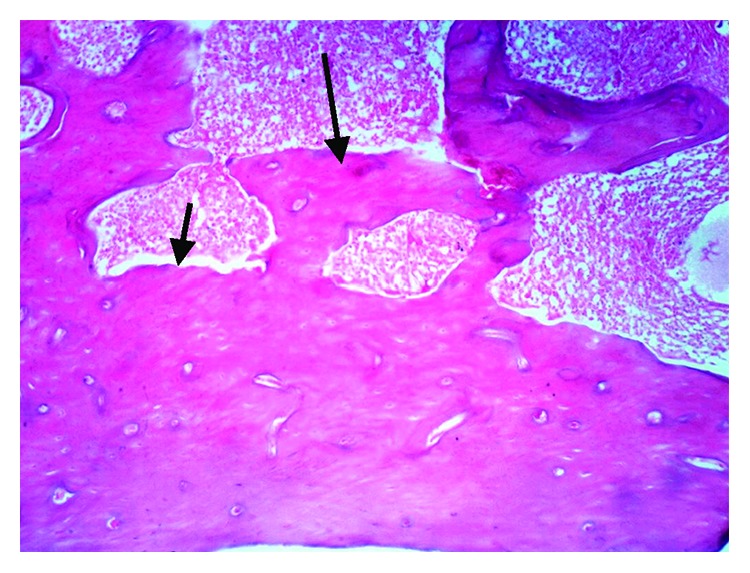
Photomicrograph of bone of rat from rosemary group (R) thick bony trabeculae with few cracks in the cortical bone (H&E X 100).

**Figure 5:**
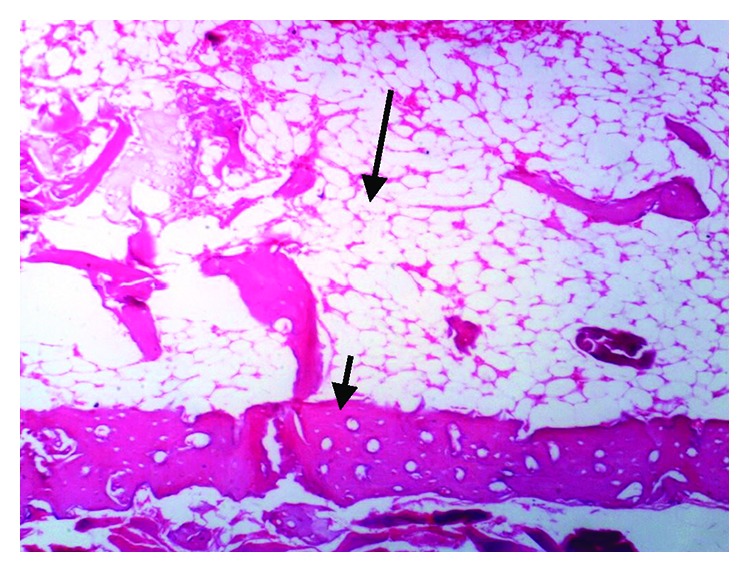
Photomicrograph of bone of rat from the positive control group (C+) showing thin cortical bone with presence of cracks and fissures as well as dilatation of marrow cavity (H&E X 100).

The histopathological examination of the femur of rats from thyme group (T) revealed evident no histopathological changes and normal bone cortex (Figure 1) as in both groups normal control (C−) (Figure 2) and oral calcium group (O) (Figure 3); otherwise, group (R) showed thick bony trabeculae with few cracks in the cortical bone (Figure 4). In contrast, several resorption cavities and thin cortical bone with presence of cracks and fissures as well as dilatation of marrow cavity were seen in the positive control group (Figure 5).

## 4. Discussion

Osteoporosis is the most widespread bone disorder, and its prevalence is expected to increase dramatically in the coming years, treatment of osteoporosis is still a big challenge, and therefore, it is seriously considered as a public health issue [29]. Calcium is an essential mineral that is connected to many metabolic interactions which provide mechanical solidity to the skeleton and teeth, where 99% of the body's calcium remains; the calcium in bones has the function of acting as a reserve supply of calcium to meet the body's metabolic requirements in the situation of calcium deficiency [30]. Calcium deficiency easily happens as a result of low calcium intake in diet and losses of calcium via the bowel and kidneys, and it may weaken the growth and delay consolidation of skeleton. Furthermore, calcium deficiency causes mobilization of bone and leads to osteoporosis and a reduction in bone minerals density [30]. The calcium requirement of human may be described as the average calcium intake needed to maintain calcium homeostasis to meet the significant obligated losses of calcium through the gastrointestinal tract, skin, and kidneys [31]. Therefore, the aim of this work was to evaluate the capability of thyme and rosemary to counteract osteoporosis and calcium deficiency on the bone microstructure, mineral content, and some biochemical markers in rats.

In our evaluation of herbs looking for active compounds for health and bone metabolism, we found that thymol was the main component of the essential oil of thyme. Some studies in Spain, Poland, and Italy reported that major compounds in thyme essential oil were thymol and *p*-cymene [32–35]. Borugă et al. [36] reported that thymol was the major component in the essential oil of *Thymus vulgaris* followed by *γ*-terpinene and *p*-cymene. Thymol is a monoterpene and considered as one of the most important dietary constituents in thyme species, and it has been used in traditional medicine; it possesses various pharmacological properties including anti-inflammatory, free-radical scavenging, analgesic, antimicrobial, and antispasmodic [37, 38]. The combination of carvacrol and thymol reduced the oxidative stress by their potent antioxidant activity [39]. It has been shown that thymol attenuates apoptosis and lipid peroxidation [40, 41]. After determination of essential oil of rosemary, we found that the main components were 1,8-cineole and camphor, and these results are in quite agreement with those reported by Boutekedjiret et al. [42] and Sienkiewicz et al. [43]. Among the herbs known for medicinal value, rosemary is highly regarded for its therapeutic potentials. *Rosmarinus officinalis* L. essential oil contains many biologically active compounds, and it confers antibacterial, anti-inflammatory, antioxidant, and pharmaceutical properties [44, 45]. Eucalyptol (1,8-cineole) has several drug properties that have developed an increasing medical interest and evidence of its anti-inflammatory and antioxidant mode of action [46]. Juergens et al. [47] found that 1,8-cineole is a strong inhibitor of cytokines with a surprising improvement in the anti-inflammatory activity.

Sapkota et al. [48] investigated the role of thymol in osteoclastogenesis and bone loss in mice and reported that thymol inhibited osteoclast activity, suppressed bone resorption, and protected against bone loss and proinflammatory cytokines; the applications of thymol significantly reduced inflammatory bone loss, and these studies identified that thymol could be a useful therapeutic agent for preventing bone serious diseases. Muhlbauer et al. [12] investigated essential oil extracted from rosemary and thyme and their monoterpene components such as eucalyptol (1,8-Cineole), camphor, borneol, thymol, *α*-pinene, and bornyl acetate; these active compounds inhibit bone resorption when added to the food of rats. The monoterpenes borneol, thymol, and camphor are direct inhibitory for osteoclast resorption, and they suggested that these compounds inhibit bone resorption by acting directly on bone cells by influencing calciotropic hormones or via stimulating the intestinal calcium absorption.

The present work indicated that the levels of plasma calcium in rats which were fed low calcium diet were significantly lower than the normal control group, and these results agree with Tannenbaum et al. [49] who recorded that the low intake levels of calcium leads to calcium deficiency in blood and increases the availability to induce osteoporosis. The obtained data suggested that the supplementation with thyme and rosemary in low calcium diet is marked by improving intestinal calcium absorption and may lead to prevent or decrease bone loss and restore the decreased levels of plasma Ca to normal values. These suggestions are in the same line with Banu et al. [50] who found that some herbs had an impact on bone metabolism including thyme and red clovers, they improved the absorption of calcium from the gastrointestinal tract, and they have an arraying effect to keep the calcium homeostasis.

Nutritional factors clearly take a part in the link between inflammation and osteoporosis, and the concordance of osteoporosis and inflammation is supported by emerging molecular evidence of mediating immunological factors [51]. Advanced understanding of the bone remodeling process proposes that factors engaged in inflammation are linked with those critical for bone physiology and remodeling [52, 53]. Furthermore, one intriguing aspect of immune ageing is the raised production of proinflammatory cytokines with osteoporosis is well documented [54]. Some inflammatory markers seen in conditions such as rheumatoid arthritis, osteoporosis, and osteomyelitis are typically associated with inflammation. Pro-osteoclastic cytokines, such as tumor necrosis factor-*α* (TNF-*α*) and interleukin-6 (IL-6), are increased in these conditions, and local cytokine profile is consistent with the cytokines that regulate bone resorption [55, 56]. There is a strong correlation between bone resorption or bone loss in healthy pre- and postmenopausal women and the production of IL-6, and TNF-*α* by peripheral blood monocytes [57]. C-reactive protein production in the liver is controlled by IL-1, IL-6, and TNF-*α* and is considered as a sensitive marker of systemic inflammation [58, 59]. A relationship between CRP level and bone mineral density has been proved in many inflammatory diseases, as well as in healthy humans, suggesting a correlation between subclinical systemic inflammation and bone loss [60]. Our results showed that thyme and rosemary have strong effect against inflammation and oxidative stress, and thyme is traditionally used for its antispasmodic and antiseptic actions. Furthermore, thyme has many activities against microbes, fungi, and viruses and also has antioxidative properties [61–63]. The essential oil of thyme is a mixture of monoterpenes, and one of the major components of this oil is thymol [64]. Thymol shows many biological properties including antioxidant [65], anti-inflammatory [66], and free-radical scavenging properties [67]. Rosemary is known to be used in traditional medicine all over the world, and the pharmacological and medical properties of rosemary include anti-inflammatory and antioxidant activities [68, 69]. Lian et al. [70] demonstrated that rosemary essential oils significantly decrease the levels of TNF-*α* in blood.

In the current study, malondialdehyde was significantly increased in the positive control rats, indicating raise in oxidative stress in these calcium-deficient rats. This result was further confirmed through the finding of elevated levels of malondialdehyde in case of bone resorption, indicating high level of free radicals generation and oxidative stress due to calcium deficiency [71]. The antioxidant of rosemary essential oils is considered as one of the herbs which contains high antioxidant properties [72], and many compounds have been isolated from rosemary, including terpenes, flavones, and steroids [73].

In addition to the improvement of bone formation, our data have shown a decrease in bone resorption as reflected by a decrease in plasma CTX levels by thyme and rosemary supplementation. Addition of thyme and rosemary had decreased CTX levels, and this response seems to be antiresorptive effect of the herb supplementation as there were elevated levels of CTX of positive control; some researches demonstrated that essential oils and their monoterpene components from common herbs and vegetables affect bone metabolism when added to the food of rats [74]. Clinical studies have shown that CTX are sensitive markers of bone loss [75, 76].

Our data showed a significant increase in vitamin D3 and decrease in PTH levels after treatment with herbs compared to positive control rats. We suggest that, with calcium deficiency in blood, the body tries to restore normal calcium levels. This often leads to an increase in the PTH level in blood, as the parathyroid glands must raise the PTH secretion in order to elevate calcium levels in blood by getting it from bones. Therefore, people with low calcium levels in blood and a high PTH levels may have secondary hyperparathyroidism, which means that the elevated level of PTH is a normal response of healthy parathyroid glands to another issue like calcium insufficiency. It is common in human to have both primary hyperparathyroidism and insufficiency of vitamin D, since primary hyperparathyroidism can lead to decreased vitamin D stores; these observations were in the same line with Berlin and Bjorkhem [77] who demonstrated that an increase in calcium intake in rats causes a reduction in the serum PTH level with a subsequent decrease in 1*α*-hydroxylation of 25-hydroxyvitamin D3, leading to raise level of 25-hydroxyvitamin D3 as a hypothetical result of suppression the action of the 25-hydroxylase. In a different study, supplementation with calcium increased the levels of 25(OH)D3 in blood significantly over 6 to 7 weeks in 28 healthy youth studied in Sweden during the winter season [78].

In the current study, diets supplemented with rosemary and thyme exhibited positive effects on BMD. The obtained results agree with the findings of Mühlbauer et al. [12] who found that bone loss was suppressed by the essential oils of these herbs; the monoterpenes and essential oil extracts act directly on bone cells through inhibition of the mevalonate pathway and the prenylation of small G-proteins such as Rac and Rho to inhibit bone resorption. Also, the positive effects of flavonoids on bone formation and BMD were studied [79, 80]. Our data showed that femur mass was decreased due to calcium deficiency in the positive control. Hunt et al. [81] found that low calcium levels significantly reduced bone mass and impaired morphology and biomechanical properties of bone in growing rats.

Our histopathological examination reflects all results obtained from biochemical parameters and femur BMD. The femur is considered to be one of the most remarkable skeletal parts in osteoporosis. The human and rat femurs have many common characters between each other at both the macrostructural and microstructural levels [82]. For these reasons, this skeletal site has got great attention and special importance in osteoporosis studies [83]. The balance between osteoblastic bone formation and osteoclastic bone resorption controls bone homeostasis. Osteoporosis occurs as a result of high bone turnover with a rate of osteoclastic resorption higher that of osteoblastic formation, resulting in a reduction of bone mass and impairment of histological characters of bones [84].

## 5. Conclusion

Our results clearly show that essential oils and monoterpenes of thyme and rosemary, which are widely used as food additives and in various medical applications, are effective inhibitors of bone resorption and have numerous benefits on bone formation and against inflammation. However, thyme has more protective effect than rosemary against bone resorption and osteoporosis.

## Figures and Tables

**Table 1:** Chemical compositions of rosemary essential oils.

Component	Kovats index	Area (%)
*α*-Pinene	937	5.63
Camphene	953	2.13
*β*-Myrcene	981	0.31
*α*-Phellandrene	990	0.50
*β*-Phellandrene	1028	1.89
Limonene	1033	1.34
1,8-Cineole	1037	46.69
Terpinolene	1100	1.39
Camphor	1153	26.30
Isoborneol	1167	0.18
Borneol	1172	0.88
Terpin-4-ol	1183	0.80
*α*-Terpineol	1196	5.23
Verbenone	1216	0.48
Bornyl acetate	1289	0.66
*β*-Caryophyllene	1432	0.50
*δ*-Cadinene	1517	2.04

**Table 2:** Chemical compositions of thyme essential oils.

Component	Kovats index	Area (%)
*α*-Pinene	937	0.27
*α*-Phellandrene	990	0.28
*α*-Terpinene	1019	0.36
*p*-Cymene	1027	4.06
*γ*-Terpinene	1061	2.49
Linalool	1099	0.45
Terpinen-4-ol	1182	0.41
*α*-Terpineol	1196	0.18
Thymol methyl ether	1235	0.33
Carvacrol methyl ether	1245	0.28
Bornyl acetate	1286	0.21
Thymol	1296	73.61
Carvacrol	1304	4.97
*β*-Caryophyllene	1432	3.33
*α*-Humulene	1470	0.38
*δ*-Cadinene	1524	0.45
*δ*-Amorphene	1531	0.65
Caryophyllene oxide	1599	3.62
Epi-*α*-cadinol	1653	1.29

**Table 3:** Plasma biochemical markers level among the different experimental groups.

Parameters	Groups				
	Group 1C−	Group 2C+	Group 3T	Group 4R	Group 5O
Ca (mg/dl)	9.62 ± 0.13a	7.57 ± 0.12d	8.92 ± 0.13bc	8.54 ± 0.11c	9.33 ± 0.12ab
P (mg/dl)	4.58 ± 0.12a	4.67 ± 0.11a	4.45 ± 0.12a	4.39 ± 0.12a	4.51 ± 0.13a
Mg (mg/dl)	1.79 ± 0.10a	1.82 ± 0.10a	2.02 ± 0.11a	1.89 ± 0.10a	1.91 ± 0.10a
MDA (*μ*mol/l)	1.33 ± 0.10d	2.42 ± 0.11a	1.92 ± 0.12b	1.85 ± 0.11bc	1.44 ± 0.10cd
TNF-*α* (pg/ml)	31.60 ± 0.94c	75.24 ± 0.93a	47.57 ± 0.96b	44.53 ± 0.98b	34.43 ± 0.79c
CRP (mg/l)	0.85 ± 0.09c	2.19 ± 0.10a	0.97 ± 0.08c	1.39 ± 0.10b	0.82 ± 0.08c
CTX (pg/ml)	320.94 ± 6.59c	507.92 ± 5.76a	379.71 ± 5.58b	392.12 ± 7.24b	339.01 ± 6.45c
Vitamin D3 (pg/ml)	64.12 ± 1.06a	46.84 ± 1.07d	55.17 ± 0.99bc	52.83 ± 0.98c	59.05 ± 1.03b
PTH (pg/ml)	45.29 ± 0.92d	69.11 ± 1.11a	50.13 ± 0.96c	58.87 ± 1.01b	41.21 ± 0.80e

All values are presented as mean ± SE. Means with different letters in each row are significantly different (*p* < 0.05).

**Table 4:** Femur mass, length, and bone mineral density of rats in different experimental animals.

Parameters	Groups				
	Group 1C−	Group 2C+	Group 3T	Group 4R	Group 5O
Femur mass (g/kg body weight)	3.02 ± 0.07a	2.46 ± 0.11b	2.93 ± 0.12ab	3.01 ± 0.17a	3.16 ± 0.07a
Femur length (mm)	33.7 ± 0.35a	32.2 ± 0.45a	32.8 ± 0.59a	31.8 ± 0.50a	33.5 ± 0.52a
BMD (g/cm2)	0.1493 ± 0.0041ab	0.0981 ± 0.0037c	0.1357 ± 0.0051ab	0.1305 ± 0.0064b	0.1525 ± 0.0051a

All values are presented as mean ± SE. Means with different letters in each row are significantly different (*p* < 0.05).

## Data Availability

The data used to support the findings of this study are available from the corresponding author upon request.
